# Sensor-to-Bone Calibration with the Fusion of IMU and Bi-Plane X-rays

**DOI:** 10.3390/s24020419

**Published:** 2024-01-10

**Authors:** Xavier Gasparutto, Kevin Rose-Dulcina, Gautier Grouvel, Peter DiGiovanni, Lena Carcreff, Didier Hannouche, Stéphane Armand

**Affiliations:** 1 Kinesiology Laboratory, Geneva University Hospitals and University of Geneva, 1205 Geneva, Switzerland; kevin.rose-dulcina@hcuge.ch (K.R.-D.); gautier.grouvel@unige.ch (G.G.); lena.carcreff@gmail.com (L.C.); stephane.armand@unige.ch (S.A.); 2 Division of Orthopaedic Surgery and Musculoskeletal Trauma Care, Surgery Department, Geneva University Hospitals and University of Geneva, 1205 Geneva, Switzerland; pcd40@georgetown.edu (P.D.); didier.hannouche@hcuge.ch (D.H.)

**Keywords:** gait analysis, IMU, fusion, sensor-to-segment calibration, bi-plane X-rays, medical imaging

## Abstract

Inertial measurement units (IMUs) need sensor-to-segment calibration to measure human kinematics. Multiple methods exist, but, when assessing populations with locomotor function pathologies, multiple limitations arise, including holding postures (limited by joint pain and stiffness), performing specific tasks (limited by lack of selectivity) or hypothesis on limb alignment (limited by bone deformity and joint stiffness). We propose a sensor-to-bone calibration based on bi-plane X-rays and a specifically designed fusion box to measure IMU orientation with respect to underlying bones. Eight patients undergoing total hip arthroplasty with bi-plane X-rays in their clinical pathway participated in the study. Patients underwent bi-plane X-rays with fusion box and skin markers followed by a gait analysis with IMUs and a marker-based method. The validity of the pelvis, thigh and hip kinematics measured with a conventional sensor-to-segment calibration and with the sensor-to-bone calibration were compared. Results showed (1) the feasibility of the fusion of bi-plane X-rays and IMUs in measuring the orientation of anatomical axes, and (2) higher validity of the sensor-to-bone calibration for the pelvic tilt and similar validity for other degrees of freedom. The main strength of this novel calibration is to remove conventional hypotheses on joint and segment orientations that are frequently violated in pathological populations.

## 1. Introduction

Human movement analysis, and specifically gait analysis, has become an essential tool to identify, characterise and follow the evolution of the locomotor system pathologies, as well as to assess the efficacy of a rehabilitation programme or surgery [1,2]. Such analysis evaluates the capacity domain of function, i.e., what a patient does in a standardised environment [3]. However, the International Classification of Function of the World Health Organisation [3] emphasises the importance of understanding the function as the interaction of multiple domains of function, including the performance, i.e., what the patient does in daily life.

The development of inertial and magnetic sensors has allowed the measurement of gait and other activities in daily life [4] as well as the development of fast tests of capacity with great potential for clinical applications [5]. Based on sensor fusion algorithms and integration, these low-cost, lightweight sensors can measure their change in orientation [6]. However, since IMUs record the change in orientation in their own coordinate system, they are blind to their environment and a calibration, known as sensor-to-segment calibration [7], needs to be performed to estimate the segment and joint kinematics during movements such as gait [8]. This calibration represents one of the main challenges of using IMUs in motion capture. Four techniques are commonly used and mixed in the literature to obtain sensor-to-segment calibration: manual, static, functional and anatomical techniques; with currently no evidence on which method shows superior results [7].

These techniques have respective limitations that can greatly limit their applicability in clinical settings, such as long measurement time, difficult protocol for patients or hypotheses that can be too strong in pathological populations. Indeed, with the manual technique, IMUs are aligned with the anatomical axis of the segment. This method relies highly on the operator’s expertise and, since anatomical axes are not orthogonal, this technique can only capture one axis with the others being rough estimations [7] thus increasing the risk of crosstalk errors [9]. With the static technique, patients are asked to stand in multiple postures with the hypothesis that anatomical axes are aligned with gravity. However, some postures can be difficult to hold for patients with musculoskeletal diseases and they may not fulfil the hypotheses depending on the segments (e.g., pelvis [8]) or pathology (e.g., crouch position [10]). With the functional technique, the patients are asked to perform standardised tasks to define the functional axis of the movements. However, pain (e.g., osteoarthritis) or lack of selectivity (e.g., cerebral palsy) can make those movements difficult and impede the quality and/or the feasibility of using such calibration. Finally, in the anatomical technique, the operator uses a specific device on which a dedicated IMU is fixed to measure the anatomical axes of the patients. This technique is rather time-consuming, limiting its clinical application, and will face the well-known shortcomings of the palpation method inherent to marker-based optoelectronic systems [11].

To tackle these issues in a clinical context, we propose introducing a novel method of sensor-to-segment calibration based on the fusion of IMUs and low-dose bi-plane X-rays: the sensor-to-bone calibration. The objective is to develop a fast and reliable method to measure bone orientation with respect to IMUs and to evaluate its validity for gait kinematics measurements. To that end, we will compare our method with two marker-based reference methods: a conventional one and one based on the fusion of optoelectronic motion capture with medical imaging. With kinematics time series being characterised by their offset and patterns, we hypothesise that this novel sensor-to-bone calibration will improve the validity in terms of offset, especially for the pelvis tilt and hip range of motion, but not in terms of kinematics patterns when compared with a conventional sensor-to-segment calibration.

## 2. Materials and Methods

### 2.1. Population

The participants of this study were 8 patients (4 females, median [IQR], age: 59 [11.5] years, height: 1.68 [0.15] m, weight: 74.5 [27] kg, BMI: 25.2 [4.6] kg/m^2^) with end-stage hip osteoarthritis undergoing total hip arthroplasty and with lower limb bi-plane X-rays before and after surgery planned in their clinical trajectory. For each patient, the analysis was performed only for the operated side and only at one visit: before surgery for three patients (1 month to 1 week before surgery, patients 6 to 8) and three months after surgery for five patients (1 to 5). Due to the small sample of patients needed for this investigation, non-parametric statistics were used in this study.

### 2.2. Measurements Protocol

During their visit, patients underwent a standard lower limb bi-plane X-ray (EOS imaging, Paris, France) while equipped with a set of reflective skin markers used in optoelectronic motion capture [12] and fusion boxes (described in Section 2.3) on the pelvis and thigh of the operated side.

After the bi-plane X-ray, the patient was brought to the motion capture lab of our institution in a wheelchair to avoid fatigue and removal of the equipment. The patient was then equipped with the remaining markers of the conventional gait model marker set [13] and with 8 IMUs (Physilog 6, Mindmaze, Lausanne, Switzerland) on the trunk, pelvis, thighs, shanks and feet. IMUs of the pelvis and thigh of the operated side were placed in the fusion boxes. More specifically, the pelvis sensor was placed on the sacrum, between the posterior superior iliac spines, whereas the thigh sensor was placed on the area less affected by soft-tissue artifacts identified by Barré et al. [14], i.e., distally and laterally along the ilio-tibial band.

Once equipped, patients performed one static standing posture and multiple gait trials along a 10 m walkway with IMUs recording angular velocity and linear acceleration at 128 Hz, while marker trajectories were recorded by a 12-camera optoelectronic motion capture system at 100 Hz (Oqus7+, Qualisys, Göteborg, Sweden). 

Synchronisation between both systems was performed by fixing all IMUs on a custom plate (Supplementary Materials S1) equipped with reflective markers and by measuring rotations of the plate with both systems. Cross-correlation was then performed between the norm of the angular velocities measured with each system to assess the delay. The delay between systems was found to increase linearly with time; thus, a synchronisation task was performed at the start and end of each measurement session. Thereby, the time delay between systems was defined for each task as a function of the recording start time.

### 2.3. Fusion Box

**Figure 1:**
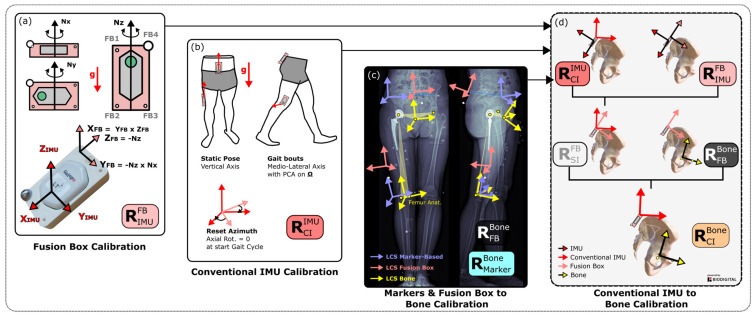
Workflow of the calibration steps for both IMU and marker-based fusion. FB stands for Fusion Box, CI for Conventional IMU approach.

The fusion box (Figure 1a) was developed to identify transformation matrices between the IMUs and the bones visible on bi-plane X-rays. In this study, only the rotation part was addressed. This box consisted of a 3D printed (UltiMaker S5, Utrecht, The Netherlands) rectangular cuboid in which the IMU was fixed (Supplementary Materials S2). Its frame contained three radio-opaque beads (2.5 mm steel bearing balls) aligned with the planes of the cuboid and a fourth and larger one (10 mm steel bearing ball) to break the symmetry on the bi-plane X-rays. First, by performing a rotation around the normal of each plane (Figure 1a), it was possible to identify the normal to the planes of the cuboid in the IMU coordinate system and thus to define the rotation matrix between the IMU and the fusion box planes ($R_{IMU}^{{FB}_{planes}}$). Then, by using the radio-opaque markers visible on bi-plane X-rays (Figure 1b), it was possible to define the normal to the planes defined by the markers in the cabin coordinate system during bi-plane X-rays and to define the orientation and position of the fusion box in the cabin coordinate system ($R_{BPXR}^{{FB}_{markers}}$). We hypothesised that the coordinate systems defined by the planes of the cuboid and by the markers were identical, thus that $R_{{FB}_{planes}}^{{FB}_{markers}}=I_{3}$. This hypothesis relied on the computer-assisted design of the box (Supplementary Materials S2) and on the submillimetre precision of the 3D printer claimed by the manufacturer.

The rotation matrix between the fusion box and the IMU ($R_{IMU}^{{FB}_{planes}}$) was defined before the patient’s arrival. Rotation around each normal to the plane of the IMUs (Figure 1a) was performed while measuring angular velocities and linear accelerations. The measured angular velocities and accelerations, respectively, defined the orientation and direction of the normal to the planes in the IMU coordinate system (***N****_x_*, ***N****_y_*, ***N****_z_*, Figure 1a). Cross-products between these axes were performed to define the orthonormal coordinate system and the rotation matrix between the IMU and the fusion box.

$$\left\{\begin{array}{c} Z_{{fb}_{planes}}={-N}_{z}\\ Y_{{fb}_{planes}}=\frac{{(-N}_{z})\;\times \;N_{x}}{\left\|{(-N}_{z})\;\times \;N_{x}\right\|}\\ \begin{array}{c} X_{{fb}_{planes}}=Y_{{fb}_{planes}}\times Z_{{fb}_{planes}}\\ R_{IMU}^{{FB}_{planes}}=\left[\begin{array}{ccc} X_{{fb}_{planes}} & Y_{{fb}_{planes}} & Z_{{fb}_{planes}} \end{array}\right] \end{array} \end{array}\right.$$

The rotation matrix between the fusion box and the bi-plane X-ray cabin (Figure 1c) was defined with the three radio-opaque markers aligned with the bottom of the cuboid. Two pairs (***FB*1**–***FB*2**, ***FB*2**–***FB*3**, Figure 1a) of these markers were aligned with the orthogonal edges of the box and a fourth bead (***FB*4**, Figure 1a) was introduced to break the symmetry and for cases where one marker was not visible. By measuring the position of these three markers in the bi-plane X-ray space, it was possible to define the normal to each plane of the cuboid as follows:

$$\left\{\begin{array}{c} \begin{array}{c} Z_{{fb}_{markers}}=\frac{FB2\;-\;FB3}{\left\|FB2\;-\;FB3\right\|}\\ y_{tmp}=FB2\;-\;FB1 \end{array}\\ \begin{array}{c} X_{{fb}_{markers}}=\frac{y_{tmp}\;\times \;Z_{{fb}_{markers}}}{\left\|y_{tmp}\;\times \;Z_{{fb}_{markers}}\right\|}\\ \begin{array}{c} Y_{{fb}_{markers}}=\frac{Z_{{fb}_{markers}}\;\times \;X_{{fb}_{markers}}}{\left\|Z_{{fb}_{markers}}\;\times \;X_{{fb}_{markers}}\right\|}\\ R_{BPXR}^{{FB}_{markers}}=\left[\begin{array}{ccc} X_{{fb}_{markers}} & Y_{{fb}_{markers}} & Z_{{fb}_{markers}} \end{array}\right] \end{array} \end{array} \end{array}\right.$$

### 2.4. Rotation Matrices of the IMU-Based Methods

#### 2.4.1. Conventional Method: Sensor-to-Segment Calibration

A method fully described in a previous study [8] was used to compute the pelvis and operated hip kinematics during gait from gyroscope and accelerometers only. It will be summarised below. 

The sensor-to-segment calibration relied on a combination of manual, static and functional techniques (Figure 1b). This proposed method was developed to be suitable for pathological subjects and clinical settings. Thus, the number and the difficulty of the instructed tasks was kept as low as possible, while still providing the same kinematic outputs as commonly provided by conventional gait analysis. The medio-lateral axis of the pelvis was defined by the axis of the IMU taped to it, one static standing pose was performed to obtain the proximal-distal axis of the pelvis and thigh, and finally, a principal component analysis was performed on the angular velocity of the thigh during gait to obtain the medio-lateral axis of this segment. These steps allowed the definition of the sensor-to-segment rotation matrices of the pelvis ($R_{{IMU}_{pelvis}}^{{Pelvis}_{CI}}$) and thigh ($R_{{IMU}_{Thigh}}^{{Thigh}_{CI}}$), *CI* standing for Conventional IMU method. Both were assumed to be constant during the movement. 

The orientation of the IMUs with respect to their initial position ($R_{{IMU}_{0}}^{{IMU}_{t}}(t)$) were estimated in the form of quaternion with a Madgwick filter (β = 0.1) [15] as a first drift correction method. To compute joint kinematics, it was assumed that all segments were aligned (flexion, rotation and adduction are set at 0°) during the static posture to allow the definition of a global frame ($R_{G}^{{IMU}_{0}}$). As a second drift correction, the orientation of the IMUs’ internal/external rotation angle was set to 0° at each foot strike. The final rotations of the pelvis and thigh with respect to the global frame and the hip kinematics were defined as follows:

$$\left\{\begin{array}{c} R_{G}^{{seg}_{CI}}\left(t\right)=R_{G}^{{IMU}_{0}}\cdot R_{{IMU}_{0}}^{{IMU}_{Seg}}\left(t\right)\cdot R_{{IMU}_{Seg}}^{{Seg}_{CI}}\\ with\;Seg\;the\;pelvis\;or\;thigh\\ R_{Hip}^{CI}=R_{{Pelvis}_{CI}}^{{Thigh}_{CI}}\;\left(t\right)={\left(R_{G}^{{Pelvis}_{CI}}\left(t\right)\right)}^{-1}\cdot \;R_{{Pelvis}_{CI}}^{{Thigh}_{CI}}\left(t\right) \end{array}\right.$$

Computations for the IMU methods were performed with quaternion formalism but, since bone coordinate systems and marker-based methods were defined from points, vectors, and matrices, this formalism was kept in the whole paper for clarity.

#### 2.4.2. Fusion Method: Sensor-to-Bone Calibration

After proper calibration of the fusion box, the orientation of the pelvis and thigh fusion boxes with respect to the bi-plane X-ray cabin coordinate system ($R_{BPXR}^{{FB}_{markers}^{Seg}}$, with Seg the pelvis or thigh) was measured based on the radio-opaque markers visible on the sagittal and frontal images (Figure 1c). Then, the bone coordinate systems of the pelvis (Anat CS1 in [16]) and thigh in [12] were defined with respect to the cabin coordinate system ($R_{BPXR}^{{Bone}_{Seg}}$) (Figure 1c). The orientation of the bone coordinate system with respect to the fusion box was then computed as follows with the hypothesis that it remains constant during the whole measurement:

$$\left\{\begin{array}{c} R_{{FB}_{markers}^{Seg}}^{{Bone}_{Seg}}={\left(R_{BPXR}^{{FB}_{markers}^{Seg}}\right)}^{-1}\cdot \;R_{BPXR}^{{Bone}_{Seg}}\\ \;\;\;\;\;\;\mathrm{with Seg the pelvis or thigh} \end{array}\right.$$

Then, the orientation of the bones with respect to the coordinate system defined with the conventional calibration of the IMUs was defined by combining the IMU calibration, the fusion box calibration and the bi-plane X-ray data as follows (Figure 1d):

$$R_{{Seg}_{CI}}^{{Bone}_{Seg}}={\left(R_{{IMU}_{Seg}}^{{Seg}_{CI}}\right)}^{-1}\cdot R_{{IMU}_{Seg}}^{{FB}_{planes}^{Seg}}\cdot R_{{FB}_{markers}^{Seg}}^{{Bone}_{Seg}}$$

Finally, this constant rotation matrix was applied as an offset correction to the outcome kinematics measured via the conventional IMU method to obtain the fusion IMU kinematics of the pelvis, thigh and hip:

$$\left\{\begin{array}{c} R_{G}^{{Seg}_{FI}}\left(t\right)=R_{G}^{{Seg}_{CI}}\left(t\right)\cdot R_{{Seg}_{CI}}^{{Bone}_{Seg}}\\ \;\;\mathrm{with Seg the pelvis or thigh}\\ R_{Hip}^{FI}=R_{{Pelvis}_{FI}}^{{Thigh}_{FI}}\;\left(t\right)={\left(R_{G}^{{Pelvis}_{FI}}\left(t\right)\right)}^{-1}\cdot R_{G}^{{Thigh}_{FI}}\left(t\right) \end{array}\right.$$

### 2.5. Rotation Matrices of the Marker-Based Methods 

#### 2.5.1. Conventional Method

The conventional gait model [13] with the hip joint centre defined by the regression of Hara et al. [17] was used for kinematic computations. This model has been abundantly described in the literature [18] and will not be further described.

#### 2.5.2. Fusion Method: Markers-to-Bone

A previously published method was used [12]. However, the pelvis coordinate was modified to the anatomical coordinate system 1 defined in Gasparutto et al. [16] to be consistent with the sensor-to-bone method described above. 

### 2.6. Segment and Joint Angles

The YXZ cardanic sequence of angles was used to decompose rotation matrices for the pelvis (anterior(+)/posterior(−) tilt, upward(+)/downward(−) obliquity, internal(+)/external(−) rotation), the thigh (anterior(+)/posterior(−) flexion, adduction(+)/abduction(−), internal(+)/external(−) rotation) and the hip (flexion(+)/extension(−), adduction(+)/abduction(−), internal(+)/external(−) rotation). Signs of the angles were then corrected to match the CGM convention. Time series were resampled between 0 and 100% for each gait cycle defined by consecutive foot strikes. Gait events were obtained automatically from IMUs of the feet [19] and were used for both IMU-based and marker-based motion capture measurements.

### 2.7. Analysis

**Figure 2:**
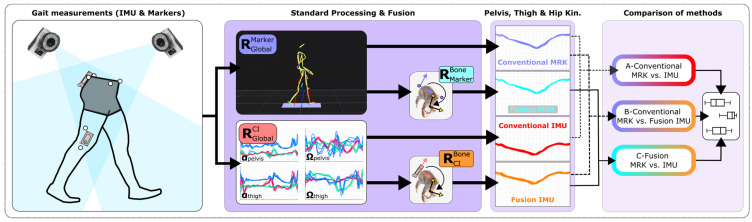
Workflow of the analysis. CI stands for Conventional IMU, Kin. for kinematics, and MRK for markers.

The root mean square difference (RMSD), the Pearson’s correlation coefficient (CC) and the absolute difference in ranges of motion (dROM) were computed for each patient and session [8]. Three validity tests were performed: (A) between the conventional markers and conventional IMUs, (B) between the conventional markers and the fusion with IMUs, and (C) between the fusion with markers and the fusion with IMU method (Figure 2). The outcomes of those tests were compared with paired Kruskall–Wallis tests and post-hoc Wilcoxon rank sum tests to evaluate the potential gains of using the fusion method for sensor-to-bone calibration (*p* < 0.05). All computations were performed with MATLAB R2021a (The MathWorks, Inc., Natick, MA, USA). 

## 3. Results

The RMSD between the fusion with markers and IMU (median [IQR], 2.8 [1.3] deg) was significantly smaller than the RMSD between the conventional marker-based and IMU-based methods (13.3 [7.8] deg) for the pelvic tilt (Table 1).

There was no difference in terms of Pearson’s Correlation Coefficient (Table 2) or in absolute differences in range of motion (Table 3) between the different validity tests.

**Figure 3:**
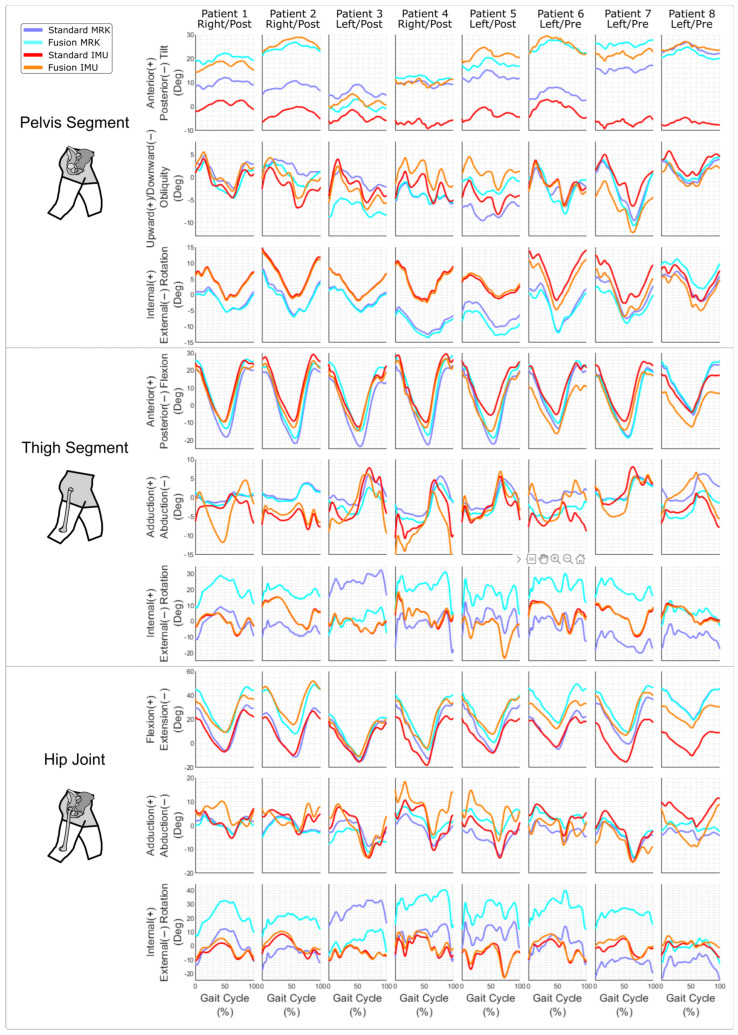
Average kinematics of the pelvis, thigh and hip with the four methods for all patients. The side of the considered hip is reported (right or left) and the visit is indicated as pre- or post-surgery. MRK stands for marker-based method, IMU stands for IMU-based method.

The kinematic timeseries of the nine degrees of freedom for the eight patients and four methods are reported in Figure 3. The range of the anterior/posterior pelvis tilt of the conventional IMU method remained between −10° and 5° across patients, whereas the other methods, including the fusion IMU, had larger inter-patient variability. The difference in internal/external rotation of the thigh and hip between the conventional and fusion IMU methods was smaller than the difference between the conventional and fusion marker methods.

## 4. Discussion

This study showed that the fusion of bi-plane X-rays and IMUs with fusion boxes to perform sensor-to-segment calibration is feasible and that it improved the validity of the pelvic tilt while keeping a similar validity on the other degrees of freedom. This calibration method could be extended to other joints or segments such as the knee, ankle, or upper limb. It could potentially be used for real-time feedback, but it would require the patients to undergo the medical imaging before the measurement session and to wait for the manual labelling of the bi-plane X-rays (10 to 20 min).

The main observed effect of the sensor-to-bone calibration was on the RMSE of the pelvic tilt. As hypothesised, this novel method allowed a proper definition of the pelvic tilt and was able to capture the variability of pelvis tilts between patients. This is specifically interesting in the context of total hip arthroplasty since the spine–hip relationship is recognised as a key factor of this surgery [20]. Assessing this variability of tilts was hardly feasible with the conventional IMU method used in this study [8] since it relied on the hypothesis that the pelvis vertical axis was aligned with gravity during static posture. This was shown on the curve with tilts across patients remaining between −10° and 5°. 

The sensor-to-bone calibration did not improve the match in terms of pelvic tilt with the clinical conventional marker-based method, i.e., the conventional gait model [13]. This seems normal since the sensor-to-bone calibration currently uses a different convention for the pelvis LCS. The bone pelvis LCS measured with bi-plane X-rays used the pubic symphysis and acetabulum whereas the marker-based LCS used the anterior and posterior superior iliac spines. It was not possible to accurately measure the anterior superior iliac spines with bi-plane X-rays [21]; thus we had to choose a different LCS definition. The question of finding a bone coordinate system of the pelvis that matches the plane typically used in motion analysis remains unanswered at this point. However, the low RMSD between the fusion IMU and fusion marker methods for the pelvic tilt (2.8 [1.3] deg) showed that our method can accurately calibrate the pelvis and that using an imaging technology that can measure iliac spines could help replicate the conventional gait model, if needed.

The method did not show any improvement of validity on the thigh sagittal angle. This suggests that the hypothesis of having the long axis of the thigh aligned with gravity during the static pose was fulfilled for this population. The reason may be that patients with hip osteoarthritis and hip replacement can usually have their lower limbs straight despite the pain. However, the sensor-to-bone calibration may be beneficial for this segment for patients that cannot fully extend their lower limb when standing (e.g., patients in crouch position [10]).

Contrary to our hypothesis, there was no improvement in terms of hip range of motion. Since the sensor-to-segment calibration impacts the pelvis tilt and thigh flexion offsets we expected a modification in terms of hip kinematics and range of motion. Our method did not improve this consistency but gave similar results to the conventional method.

The internal/external hip and thigh rotations present discrepancies in term of offset between the marker-based conventional and fusion methods. This is due in part to the definitions of LCS; the conventional method is based on the hip joint centre (estimated from pelvis markers [17]), palpated epicondyles and thigh wand marker, whereas the fusion method is based on the condyles and femoral head measured on the bi-plane X-rays. This offset was not observed between the conventional and fusion IMU, which is likely due to the drift correction that sets the IMUs’ internal/external rotation angle at 0° at each foot strike. This suggests that the hypothesis of zero rotation angle at each foot strike may be too strong and that this drift correction should be modified to avoid impeding the potential benefits of the fusion method on this degree of freedom.

Future work should also focus on going beyond this drift-correction hypothesis. The sensor-to-bone method was built on top of a conventional method based on static pose and functional calibration during gait. This conventional method allowed us to define a common global frame for the IMUs and to correct the drift at each step. The sensor-to-bone method could be independent from it with either a specific external device creating a global frame for the IMU combined with specific drift-free integration algorithm, and/or with the additional use of magnetometers to define a common global coordinate system for the IMUs.

As hypothesised, the sensor-to-bone calibration did not have any effect on the coefficient of correlation. Indeed, this new method only applies an offset to the kinematics computed with the conventional IMU method. Thus, it did not impact the patterns of the time series and will not improve this aspect of validity or accuracy. Different sensor fusion algorithms and/or IMU placements should be tested to improve the consistency of the time series’ pattern between marker-based and IMU-based methods.

It should be noted that the sensor-to-bone calibration by the fusion method does not take soft-tissue artifacts into account. Indeed, it relies on the hypothesis of a constant rotation between the IMU and the underlying bones which is known to be violated during movements [22]. However, this sensor-to-bone calibration has the potential to measure position from an IMU to any measurable anatomical point. Thus, it could be used to define STA compensation algorithms such as patient-specific kinematics chains based on medical imaging that may help in improving the validity and accuracy of IMU-based motion capture. 

Finally, this method implies the use of medical imaging and thus should be restricted to patients that undergo such exams during their clinical pathway to keep the irradiations at a reasonable level. Indeed, the gain in term of pelvis calibration may not balance the increased radiation dose. Inclusion or exclusion of patients for this method will not be discussed here, since the authors believe that prescribing medical imaging and determining its modality should remain with the clinical team caring for the patient. An additional limitation for bi-plane X-rays is the maximal field of view of the bi-plane X-rays (0.34 m on frontal view and 0.32 m on sagittal view at the centre of cabin). This can be narrow for adults and the lateral fusion box may be out of the field. To solve this issue, patients were asked to have the internal part of both feet along the centre line of the cabin. 

This study focused on the validity of the methods; however, the reliability is a critical point, especially when dealing with longitudinal assessments of function. The reliability of the axis definition will rely on five main sources of errors: (1) the 3D printing of the fusion box, (2) the manual placement of the steel beads in the fusion box, (3) the manual calibration of the fusion boxes with the rotations on the different normal to the fusion box, (4) the identification of anatomical landmarks on bi-plane X-rays and (5) the identification of the steel beads on the bi-plane X-rays. Among these sources, only the identification of anatomical landmarks was evaluated clearly with the smallest detectable changes between 1 mm and 5 mm. Since the accuracy of the radio-opaque implants was very high, the accuracy of the position of the steel beads is expected to be below 1 mm. The 3D printing, placement of steel beads and calibration of fusion boxes will depend mainly on the accuracy of the 3D printing that was reported below 1 mm. Conversely, the reliability of sensor-to-segment methods will rely on the repeatability of a patient’s posture and movement between sessions. A previous study on the fusion of motion capture and bi-plane imaging showed that the fusion method could reduce marker misplacement [12], the main source of inter-session variability [11], and that it should improve inter-session reliability to the level of bi-plane X-ray reliability [21]. Thus, we hypothesise that the sensor-to-bone calibration with the fusion technique may increase the reliability of IMU-based motion capture and prove useful for longitudinal assessment of function. This hypothesis should be tested in future studies and completed with an evaluation of the sensitivity to change of the method. 

In summary, the main strength of the sensor-to-bone calibration method is that it goes beyond three hypotheses on which conventional IMU calibration methods rely: (1) the hypothesis that segments are aligned with gravity in the static pose, (2) the hypothesis that the internal–external rotations are null in the static pose, and (3) that a patient’s gait is mostly in the sagittal plane. Indeed, these hypotheses may not hold when assessing patients with joint stiffness, pain or bone deformities. However, since the drift correction relies on setting internal–external rotation angles to zero at foot strike, the present iteration of the method could not remove this hypothesis.

## 5. Conclusions

The sensor-to-bone calibration with the fusion of IMUs and bi-plane X-rays is feasible and allows the proper definition of the anatomical axis, specifically for the pelvis tilt. The main strength of this novel calibration method is that it goes beyond classical hypotheses on joint and segment orientations that are frequently violated in populations with musculoskeletal disorders. For patients within the boundaries of these hypotheses, the method will show similar validity, but for conditions outside of these hypotheses, the validity should be improved. Nevertheless, this method requires medical imaging and should be used for patients undergoing imaging in their clinical pathway to limit radiation exposure.

## Figures and Tables

**Table 1:** Root Mean Square Difference in degrees as median [Interquartile Range]. *MRK* stands for marker-based method, IMU stands for IMU-based method.

	Dof	A—Conventional MRK vs. IMU	B—Conventional MRK vs. Fusion IMU	C—Fusion MRK vs. IMU	Kruskall–Wallis (*p*-Value)	Post-Hoc Wilcoxon
Pelvis	Tilt	13.3 [7.8]	6.9 [8.2]	2.8 [1.3]	0.004	A > C
	Obliquity	2.3 [0.8]	3 [3]	2.8 [1.8]	0.833	-
	Rotation	6.5 [4]	6.4 [4.4]	7 [4.1]	0.779	-
Thigh	Flexion	8.6 [3.1]	7.3 [1.8]	5.5 [1.8]	0.33	-
	Add-Abd	6.1 [3]	6.7 [1.7]	7 [1]	0.728	-
	Rotation	11.4 [5.1]	11.4 [5.2]	18.1 [8.7]	0.545	-
Hip	Flex-Ext	7.2 [7.1]	10.8 [4.5]	5.8 [2]	0.181	-
	Add-Abd	4.6 [2.6]	5.5 [2.6]	6 [1.5]	0.493	-
	Rotation	11.1 [5.4]	11.9 [6.7]	24.7 [13.1]	0.183	-

**Table 2:** Pearson’s Correlation Coefficient as median [Interquartile Range]. MRK stands for marker-based method, IMU stands for IMU-based method.

	Dof	A—Conventional MRK vs. IMU	B—Conventional MRK vs. Fusion IMU	C—Fusion MRK vs. IMU	Kruskall–Wallis (*p*-Value)	Post-Hoc Wilcoxon
Pelvis	Tilt	0.6 [0.2]	0.7 [0.3]	0.6 [0.3]	0.968	-
	Obliquity	0.8 [0.2]	0.8 [0.2]	0.8 [0.2]	0.935	-
	Rotation	0.9 [0.1]	0.8 [0.1]	0.8 [0.1]	0.636	-
Thigh	Flexion	1 [0]	1 [0]	1 [0]	0.968	-
	Add-Abd	−0.3 [0.5]	−0.6 [0.4]	−0.6 [0.4]	0.761	-
	Rotation	0.4 [0.3]	0.4 [0.3]	0.4 [0.3]	0.954	-
Hip	Flex-Ext	1 [0]	1 [0]	1 [0]	0.998	-
	Add-Abd	0.8 [0.4]	0.5 [0.6]	0.5 [0.6]	0.221	-
	Rotation	0.3 [0.2]	0.3 [0.2]	0.3 [0.2]	0.086	-

**Table 3:** Absolute difference in range of motion in degrees as median [Interquartile Range]. MRK stands for marker-based method, IMU stands for IMU-based method.

	Dof	A—Conventional MRK vs. IMU	B—Conventional MRK vs. Fusion IMU	C—Fusion MRK vs. IMU	Kruskall–Wallis (*p*-Value)	Post-Hoc Wilcoxon
Pelvis	Tilt	1.3 [0.8]	1.1 [0.8]	0.9 [0.6]	0.914	-
	Obliquity	2 [2.1]	2 [2]	2.6 [1.7]	0.998	-
	Rotation	2.1 [1.4]	2.2 [1.3]	2.4 [1.6]	0.578	-
Thigh	Flexion	5.4 [2.1]	6.3 [2.8]	6.5 [2.7]	0.573	-
	Add-Abd	4.7 [3.4]	6.3 [3.3]	6.1 [4.2]	0.255	-
	Rotation	5 [3.3]	4.5 [3.7]	4.6 [3.6]	0.968	-
Hip	Flex-Ext	4.2 [2.3]	5.3 [3.6]	5.7 [3.3]	0.566	-
	Add-Abd	6.8 [3.8]	6.8 [7.6]	6.9 [7.6]	0.846	-
	Rotation	8.2 [7.7]	8.8 [8.7]	9.2 [9.2]	0.878	-

## Data Availability

The data presented in this study are openly available in Yareta at https://doi.org/10.26037/yareta:43qzybuvanapnch374a2us3p7a.

## Supplementary Materials

The following supporting information can be downloaded at: https://www.mdpi.com/article/10.3390/s24020419/s1, Supplementary Materials S1: Computer-assisted design of the plate used to synchronise the optoelectronic motion capture system and the inertial measurements units. Supplementary material S2: Computer-assisted design of the fusion box.
